# Pruriplastic Itch—A Novel Pathogenic Concept in Chronic Pruritus

**DOI:** 10.3389/fmed.2020.615118

**Published:** 2021-01-20

**Authors:** Laurent Misery

**Affiliations:** Univ Brest, LIEN, Brest, France

**Keywords:** pruritus, psychogenic, sensitization, itch, neuropathic

## Abstract

The International Association for the Study of Pain (IASP) defined three descriptors for pain: nociceptive pain is “pain that arises from actual or threatened damage to non neural tissue and is due to the activation of nociceptors”; neuropathic pain is “pain caused by a lesion or disease of the somatosensory nervous system”; and nociplastic pain is “pain that arises from altered nociception despite no clear evidence of actual or threatened tissue damage causing the activation of peripheral nociceptors or evidence for disease or lesion of the somatosensory system causing the pain.” Based on clinical and pathophysiological arguments, a similar definition of “pruriplastic pruritus” should be made. Pruriplastic pruritus would include psychogenic pruritus, as well as some cases of pruritus ani, vulvar pruritus, sensitive skin or other poorly understood cases of pruritus. This new descriptor of itch could serve as systematic screening for altered pruriceptive function in patients who suffer from chronic itch and it may also help in defining better tailored treatment by identifying patients who are likely to respond better to centrally rather than to peripherally targeted therapies.

## Introduction

In 2007, the International Forum for the Study of Itch (IFSI) proposed a classification of chronic itch based on clinical signs and distinguished between diseases with and without primary or secondary skin lesions (1). Three groups of conditions were proposed: pruritus on diseased (inflamed) skin (group I), pruritus on non-diseased (non-inflamed) skin (group II), and pruritus presenting with severe chronic secondary scratch lesions, such as prurigo nodularis (group III). The next part classified the underlying diseases according to different categories: dermatological diseases, systemic diseases including diseases of pregnancy and drug-induced pruritus; and neurological and psychiatric diseases. In some patients, more than one cause may account for pruritus (category “mixed”), while in others, no underlying disease can be identified (category “others”). Thus, it was concluded that our knowledge regarding neurophysiological and pathophysiological aspects of chronic pruritus has developed and that this initial classification will undoubtedly require future revisions.

Patients with pruritic diseases of systemic, neurological or psychosomatic/psychiatric origin should be included in group II or group III. It was recommended that the term pruritus sine materia should no longer be used because this term has multiple interpretations, such as idiopathic pruritus, pruritus without any skin changes, pruritus in systemic diseases without any initially visible skin changes, pruritus characterized by the absence of specific cutaneous lesions of an itching dermatosis, pruritus in the elderly or psychosomatic pruritus (1).

Indeed, there is a psychosomatic definition of pruritus sine materia in the ICD-10 classification of F45.8 as somatoform pruritus, which was considered the more acceptable term by the IFSI (1). Neuropathic itch should refer to pruritus caused by neuronal or glial damage (2), whereas psychogenic itch is related to psychological disorders (3). Nonetheless, the concept of somatoform or psychogenic itch remains controversial.

## Controversies Related to Psychogenic Itch

Psychogenic itch is known as psychogenic pruritus, somatoform pruritus (4), functional itch disorder (5), non-organic pruritus, psychosomatic pruritus, or functional pruritus, but “psychogenic itch” is the most commonly used denomination (6).

The 11th revision of the International Classification of Diseases (ICD-11) clearly separates psychogenic pruritus (EC90.4, using this term), from pruritus of unknown origin (EC90.6) among other causes of pruritus (EC90).

European guideline on chronic pruritus proposes that somatoform pruritus is defined as pruritus where psychic, psychiatric and psychosomatic factors play a critical role in the onset, intensity, aggravation or persistence of the pruritus (7).

The French Psycho-Dermatology Group (FPDG) is an expert group from the French Society of Dermatology that includes dermatologists, psychologists and psychiatrists. This group has proposed a definition of psychogenic pruritus as “an itch disorder where itch is at the center of the symptomatology and where psychological factors play an evident role in the triggering, intensity, aggravation or persistence of the pruritus” and has suggested calling it “functional itch disorder” (FID) (5). The FPDG also proposed 10 diagnostic criteria (3 compulsory and 7 optional) to assess the diagnosis, with both negative (no somatic cause) and positive criteria (clinical characteristics, association with psychological disorders or stressful life events).

Previous psycho-dermatological classifications (associated skin and psychological disorders) had included psychogenic pruritus among the “psychological disorders responsible for skin sensations” (8), “functional cutaneous and mucous disorders” (9) or “conditions in which strong psychogenic factors are imputed” (10). It is of particular interest to separate psychogenic excoriations (11), dermatitis artefacta and all other self-inflicted skin lesions (SISLs) (12) from psychogenic pruritus. SISLs are related to impulsive, compulsive or other psychopathological mechanisms. In contrast, psychogenic pruritus is related to an illusion of pruritus, but this pruritus is felt by the patient and is the main complaint.

Psychogenic pruritus was not precisely cited in the 4th edition of the Diagnostic and Statistical Manual of Mental Disorders (DSM-IV) (13), but it could be recognized among the following 4 diagnoses (6) listed in the DSM-IV:

- Conversion disorder (300.11): Unexplained symptoms or deficits affecting voluntary motor or sensory function that suggest a neurological or other general medical condition. Psychological factors are judged to be associated with the symptoms or deficits.
- Undifferentiated somatoform disorders (300.81): One or several somatic complaints lasting 6 months or more with no medical or mental disease available to explain the presence or intensity of these symptoms. This symptom is not intentionally self-induced or simulated.
- Unspecified somatoform disorder (300.82): All disorders with somatoform symptoms that do not fit the criteria of any specific somatoform disorder.
- Pain disorder associated with psychological factors (307.80): Psychological factors play a critical role in the triggering, intensity, aggravation or persistence of the pain.

The 5th edition of the Diagnostic and Statistical Manual of Mental Disorders (DSM-5) (14) defined a new diagnosis of “somatic symptom disorder” (SSD), which is characterized by “somatic symptoms that are either very distressing or result in significant disruption of functioning, as well as excessive and disproportionate thoughts, feelings and behaviors regarding those symptoms. To be diagnosed with SSD, the individual must be persistently symptomatic (typically for at least 6 months).”

The DSM-IV disorders of somatization disorder, hypochondriasis, pain disorder, and undifferentiated somatoform disorder were removed. The DSM-IV diagnosis of somatization disorder required a specific number of complaints from among four symptom groups while the SSD criteria must only be significantly distressing or disruptive to daily life and must be accompanied by excessive thoughts, feelings, or behaviors. While medically unexplained symptoms were a key feature for many of the disorders in the DSM-IV, a diagnosis of SSD does not require that the somatic symptoms are medically unexplained. Consequantly, symptoms may or may not be associated with another medical diagnosis. Furthermore, it is not appropriate to diagnose individuals with a mental disorder solely because a medical cause cannot be demonstrated. Regardless of whether the somatic symptoms can be medically explained, the individual would still have to meet the remaining criteria to receive a diagnosis of SSD.

As in the case of psychogenic pain (15), the concept of SSD overpsychologizes people with chronic pain and may contribute to misdiagnosis and unnecessary stigma (16). In contrast, the DSM-5 SSD criteria may be more restrictive than the DSM-IV criteria for somatoform disorders for a population of patients with medically unexplained physical symptoms (MUPS) (17).

Because SSD include both psychogenic pruritus and pruritus of a somatic origin, this disproportionate resounding is very confusing (6). Nonetheless, we have to admit that it is very difficult to separate in clinical practice patients with a pure psychogenic pruritus from those with a large but not exclusive contribution of psychological factors in the pathogenesis of their pruritus. Numerous psychological factors could influence the perception and modulation of pruritus (18–24).

## Unified Pathophysiology

In case of pruritus or even if we think about pruritus or scratching sensory, motor and affective areas of the brain are activated (25–28). Consequently, a new definition of pruritus could be “a sensation accompanied by the contralateral activation of the anterior cortex and the predominantly ipsilateral activation of the supplementary motor areas and the inferior parietal lobule; scratching may follow” (29), reflecting the fact that “it is the brain that itches, not the skin” (30). Hence, a psychological component may occur in all cases of pruritus (31) and that a specific psychogenic pruritus can exist (30).

Convergent visceral-somatic processing and neuroimaging studies in somatoform disorders have demonstrated that somatosensory amplification may, in part, develop through stress-mediated neuroplastic changes and the neuromodulatory effects of inflammation (32). Neural correlates of cognitive-affective amplifiers are integrated into a network for somatosensory amplification, including anterior cingulate cortex, insula, amygdala, hippocampal formation, and striatum.

Transient scratching inhibits the itch sensation, but repeted and increasing scratching is involved in peripheral and central sensitizations to itch (30, 33–35). Similarly to pain sensitizations, inflammatory mediators are released by pruriceptors (peripheral sensitization), whereas chronic skin inflammation facilitates spinal and cerebral itch processing, resulting in touch-evoked pruritus (central sensitization). Clinical consequences are alloknesis and hyperknesis (36), which could be confused with psychological involvement in itch, extending up to itch catastrophizing.

All humans can suffer from itch in the course of their life (37, 38). Witnessing your neighbor scratching, discussing or reading about itch, watching movies showing people scratching and viewing pictures of affected skin or insects can induce itch in healthy persons and to a greater degree in chronic itch patients and subjects with neurotic personalities (39). The underlying course of contagious itch seems to be related to human mirror neurons that are active when we imitate actions and/or negative affects (38). Brain imaging evidenced an important functional coupling of the insula and basal ganglia in initiating the urge to scratch when provided with itch-evoking visual stimuli (40).

Several studies suggest that mechanisms of central modulation play an important role in the development and maintenance of chronic itch (41). The management of the neurosensory aspects of itch is an important part of the management of chronic itch. The reason for our underlying desire to itch may be an overactive limbic system, particularly the anterior cingulate cortex, which is essential in modulating emotional and cognitive activities (39). The co-activation of the prefrontal cortex in conjunction with the limbic system upon application of itch stimuli suggests interplay of this network on motivation and emotion (42). The role of the prefrontal cortex has been highlighted in reward processing for addictive behaviors (39). The reward circuit, particularly in the midbrain, may be part of the neural basis of the vicious itch-scratch-itch circle and appears to be linked to the development of an urge to scratch to achieve the “pleasure” derived from scratching (40). The involvement of the midbrain strongly suggests a role for the dopaminergic system in the addictive nature such a circle (43). Central mechanisms of itch have been previously reviewed (41, 44–46). More recently, the growing number of brain imaging studies has allowed meta-analyses (47, 48).

In addition to clinical considerations, pathophysiological data on the brain processing of itch support that it not possible to separate psychogenic from non psychogenic itch.

## Lessons From Pain Research

Because mechanisms of chronic itch are close to those of chronic pain (36, 44, 49–52) or chronic cough (50, 53), it is very interesting to observe the evolution of thoughts in these fields.

The first definition for neuropathic pain was given in 1994 by the International Association for the Study of Pain (IASP) as “pain initiated or caused by a primary lesion or dysfunction in the nervous system.” This definition was changed to a new one in 2005 when the nociceptive terminology appeared. Nociceptive pain was defined as “pain due to stimulation of primary nociceptive nerve endings,” and neuropathic pain was defined as “pain due to lesion or dysfunction of the nervous system” (54). Consequently, we could modify the definition of neuropathic pruritus (2). Both definitions are periodically reviewed, and currently, nociceptive pain is “pain that arises from actual or threatened damage to nonneural tissue and is due to the activation of nociceptors,” and neuropathic pain is “pain caused by a lesion or disease of the somatosensory nervous system” (54).

Because this dichotomy between pain mechanistic definitions created a gap for numerous patients without activation of neither nociceptors nor lesion or disease of the nervous system, a third descriptor was proposed in 2016 (55) then adopted by the IASP council in 2017, although it is not yet universally agreed: nociplastic pain. This choice was supported by abundant literature confirming changes in cerebral activation in once-called “dysfunctional diseases” (56, 57) and changes in cerebral connectivity across multiple chronic pain conditions (58). More recent studies have shown that central pain processing is augmented after psychological trauma (alterations in painful and non-painful areas), whereas there are only alterations in the painful area without such a trauma (59). The relationship between anxiety or depression symptoms, and pain intensity is completely mediated by central sensitization while the relationship between catastrophic thinking and pain intensity is partially mediated by central sensitization (60).

The chosen definition of nociplastic pain is “pain that arises from altered nociception despite no clear evidence of actual or threatened tissue damage causing the activation of peripheral nociceptors or evidence for disease or lesion of the somatosensory system causing the pain” (55).

Patients can have a combination of nociceptive and nociplastic pain (55). The concept of mixed pain, which is defined as “a complex overlap of the different known pain types (nociceptive, neuropathic, and nociplastic) in any combination, acting simultaneously and/or concurrently to cause pain in the same body area. Either mechanism may be more clinically predominant at any point in time. That mixed pain can be acute or chronic” has also been proposed (61).

## Conclusions

Hence, it should be proposed to use three descriptors for itch (Table 1):

- Pruriceptive itch: itch that arises from actual or threatened damage to non-neural tissue and is due to the activation of pruriceptors
- Neuropathic itch: itch caused by a lesion or disease of the somatosensory nervous system
- Pruriplastic itch: itch that arises from altered pruriception despite no clear evidence of actual or threatened tissue damage causing the activation of peripheral pruriceptors or evidence of disease or lesions of the somatosensory system causing the itch
- Mixed itch: overlap of the different known itch types (nociceptive, neuropathic, and nociplastic) in any combination, acting simultaneously and/or concurrently to cause itch in the same body area.

**Table 1:** Comparisons between pruriceptive, neuropathic and pruriplastic itch.

	**Pruriceptive**	**Neuropathic**	**Pruriplastic**
Origin	Pruriceptor	Lesions of peripheral or central nervous system	Dysfunctions of itch processing (central sensitization, loss of downstream controls)
Characteristics	Variables	Associated with other unpleasant sensations (paresthesia, dysesthesia)	Atypical^*^
Localization	Locoregional or diffuse	According to involved nervous system	Local or diffuse
Neurological examination	Normal	Abnormal	Normal
Evolution	Acute or chronic	Chronic	Chronic
Treatment	Etiological Symptomatic	Gabapentinoids Antidepressants^**^	Antidepressants^**^

** *There is a need for confirmation by clinical trials*.

* *Examples: association with extra-cutaneous sensory or other disorders*.

Pruriceptive and neuropathic pruritus are not new concepts but pruriplastic pruritus is a new one. Pruriplastic pruritus would include psychogenic pruritus, as well as some cases of pruritus ani, vulvar pruritus, sensitive skin or other poorly understood cases of pruritus. It would be the pruritic equivalent of nociplastic pain, including vulvodynia, burning mouth syndrome, fibromyalgia and all SSDs.

In clinical practice, the diagnosis of pruriplastic pruritus could be a neutral, more acceptable and understandable diagnosis for patients with these disorders, avoiding oppositions between neurogenic and psychogenic hypotheses. Indeed, many patients are afraid by psychogenic hypotheses or refuse diagnoses of psychogenic diseases because that challenges them personally. In the absence of direct evidence of skin or somatosensory disorders, it would be useful to have a category of “pruriplastic itch” to reassure the patient that their itch is real.

Finally, it could be used for further clinical research and diagnostic criteria might be defined. Like nociplastic pain (54), this new descriptor of itch could serve as systematic screening for altered pruriceptive function in patients who have chronic itch and it may also help in defining better tailored treatment by identifying those who are likely to respond better to centrally rather than to peripherally targeted therapies.

Like nociplastic pain, there is likely an organic basis for pruriplastic itch such as central sensitization of itch pathways, although central sensitization may also occur in all causes of chronic itch, in the absence of overt evidence of disease or lesions of the somatosensory system. The loss of descending pathways has been recently evidenced as a hallmark of chronic itch patients, who have reduced conditioned pain modulation (CPM) which might contribute to enhanced itch (62). Otherwise there is little clinical evidence based on quantitative sensory testing (QST) to suggest that chronic itch patients exhibit altered pruriception (e.g., alloknesis or hyperknesis). This proposal would be strengthened by including any studies that have investigated pruriception in patients suffering from chronic itch of different origins including psychogenic/pruriplastic itch.

Hence, I propose this new concept of pruriplastic itch, which needs discussion by colleagues and a final validation by the IFSI.

## Data Availability Statement

The raw data supporting the conclusions of this article will be made available by the authors, without undue reservation.

## Author Contributions

The author confirms being the sole contributor of this work and has approved it for publication.

## Conflict of Interest

The author declares that the research was conducted in the absence of any commercial or financial relationships that could be construed as a potential conflict of interest.
